# Unobtrusive Sensors for the Assessment of Older Adult’s Frailty: A Scoping Review

**DOI:** 10.3390/s21092983

**Published:** 2021-04-23

**Authors:** Antonio Cobo, Elena Villalba-Mora, Rodrigo Pérez-Rodríguez, Xavier Ferre, Leocadio Rodríguez-Mañas

**Affiliations:** 1 Centre for Biomedical Technology (CTB), Universidad Politécnica de Madrid (UPM), Pozuelo de Alarcón, 28223 Madrid, Spain; xavier.ferre@ctb.upm.es; 2 Centro de Investigación Biomédica en Red en Bioingeniería, Biomateriales y Nanomedicina (CIBER-BBN), 28029 Madrid, Spain; 3 Fundación para la Investigación Biomédica del Hospital Universitario de Getafe, Hospital de Getafe, Getafe, 28905 Madrid, Spain; rprodrigo@salud.madrid.org; 4 Servicio de Geriatría, Hospital de Getafe, Getafe, 28095 Madrid, Spain; leocadio.rodriguez@salud.madrid.org; 5 Centro de Investigación Biomédica en Red de Fragilidad y Envejecimiento Saludable (CIBER-FES), 28029 Madrid, Spain

**Keywords:** unobtrusiveness, ubiquity, transparency, sensors, frailty syndrome, older people, smart home

## Abstract

Ubiquity (devices becoming part of the context) and transparency (devices not interfering with daily activities) are very significant in healthcare monitoring applications for elders. The present study undertakes a scoping review to map the literature on sensor-based unobtrusive monitoring of older adults’ frailty. We aim to determine what types of devices comply with unobtrusiveness requirements, which frailty markers have been unobtrusively assessed, which unsupervised devices have been tested, the relationships between sensor outcomes and frailty markers, and which devices can assess multiple markers. SCOPUS, PUBMED, and Web of Science were used to identify papers published 2010–2020. We selected 67 documents involving non-hospitalized older adults (65+ y.o.) and assessing frailty level or some specific frailty-marker with some sensor. Among the nine types of body worn sensors, only inertial measurement units (IMUs) on the waist and wrist-worn sensors comply with ubiquity. The former can transparently assess all variables but weight loss. Wrist-worn devices have not been tested in unsupervised conditions. Unsupervised presence detectors can predict frailty, slowness, performance, and physical activity. Waist IMUs and presence detectors are the most promising candidates for unobtrusive and unsupervised monitoring of frailty. Further research is necessary to give specific predictions of frailty level with unsupervised waist IMUs.

## 1. Introduction

Disability is one of the major challenges for elderly care. Even though people live longer, they are expected to spend many years dealing with disability [1]. For example, the forecast for trends in England and Wales predicts an increase in life expectancy with disability at age 65 from 4.7 years in 2015 to 5.4 years in 2025 [1]. Conversely, a successful aging path would delay the onset of disability until very close to the end of life [2]. Disability may be preceded by several years by a state of increased vulnerability known as frailty [3]. Frailty is a multidimensional concept involving different biological systems (nervous, endocrine, immune, and musculoskeletal) [4]. It makes homeostasis difficult even when a frail person is exposed to low power stressors [4]. Frailty places older people at high risk of adverse outcomes, including twice the risk of disability of non-frail older adults [5], as well as falls, hospitalization, permanent institutionalization, and death [4,6,7,8]. The role of the nervous/cognitive system and others in the “frailty cycle” has been recognized since the earliest pathophysiological theories of frailty [9]. The links between frailty and cognition are widely recognized, but this relationship does not mean admitting the existence of cognitive frailty, just as the existence of vascular pathways for frailty does not mean the existence of a vascular subtype of frailty. Frailty is an end point of many different pathways (e.g., similar to the different aetiologias of heart failure—ischemic, cardiomyopathic, etc.) to allow for underlying social, cognitive, physical, etc., causes, but none of these, taken one by one, are able to produce frailty [10]. There are other conditions strongly related to disability. In particular, Parkinson’s disease (PD) is very disabling, but disability due to PD is not necessarily preceded by frailty. The consequences of falls may be very disabling as well, and there are many studies focused on fall detection, fall prevention, fall prediction, prediction of the risk of falls, etc. However, even though there is known to be a connection between falls and frailty, not every recurrent faller is frail. Finally, cognitive issues (such as cognitive impairment, dementia, or Alzheimer’s Disease) strongly deteriorate people’s independence, but the present review focuses on definitions of frailty that do not consider cognitive impairment.

There are two major approaches to model frailty. On the one hand, Rockwood’s deficit accumulation model defines the frailty index (FI) as the number of health deficits observed in an individual divided by the total number of health variables under study [11,12,13]. On the other hand, Fried’s phenotypic model has attracted a lot of attention and defines frailty as a clinical syndrome that can be diagnosed by assessing five variables, namely, slowness, weakness, exhaustion, weight loss, and low physical activity [3]. Fried’s phenotype defines three levels of functional status. First, frail people are those at high risk of developing disability. Any older adult testing positive for any three of the five functional variables in Fried’s phenotype is frail. Second, pre-frail people are at lower risk of developing disability than frail people. Any older adult testing positive to any one or two of the five variables in the phenotype is pre-frail. Finally, robust people are those at low risk of developing frailty. Robust people do not test positive to any of the variables in the phenotype. 

Fortunately, in contrast to disability, frailty can be reversed [14,15,16]. Clinical interventions based on physical exercise have been observed to reverse frailty [14,15,17]. These exercise-based interventions are particularly effective if frailty is diagnosed at early stages of the functional decline process and the older adult remains engaged to the care program [18]. Even though monitoring the progression of functional decline in frail people is required to measure the effectiveness of the interventions and adapt them accordingly, monitoring the functional status of robust people is also important to detect the onset of frailty early and apply early interventions. The progressive process of physiological decline that takes people from robustness to frailty and ends up in dependency is associated with old age. The border between adult and older age is fuzzy, with different countries and organizations applying different criteria. In Spain, people are considered as older adults as they turn 65, which used to match the legal age for retirement. However, people requiring functional recovery interventions in geriatrics departments such as the Hospital of Getafe’s are usually older than that (70+ years old). Thus, we have considered monitoring people from 65 years old on enough to look for early signs of frailty. 

Currently, older people are not screened for early signs of functional decline, because the assessment of frailty requires the participation of trained professionals in a geriatrics department and is time-consuming. In fact, the role of geriatrics departments, as part of specialized care, is to take care of uncontrolled cases with values for their clinical markers beyond regular boundaries. Automatic sensors that do not require the involvement of any specifically trained personnel have been proposed as a tool for older adults to monitor their functional status at their homes [19]. The most obvious solution would be to have one or more sensors able to measure the values for the variables in the Fried’s phenotype and then apply Fried’s criteria to determine the subject’s frailty level. Another approach would be to develop sensors able to assess whether a subject is robust, pre-frail, or frail even without necessarily computing any partial frailty markers or combining them in a composite score. In addition to these variables, geriatricians also use another category of variables known as measurements of physical performance, such as the Timed Up and Go (TUG) test [20] and the Short Physical Performance Battery (SPPB) test [21] to assess functional decline in older people. Throughout the paper, we have collectively referred to all of these variables (frailty level, Fried’s variables, and performance) as functional variables, because the different values they can take describe the status of the subjects’ overall function (frailty level) or some of its partial features (Fried’s variables and performance). However, the vast majority of the older population are not technologically savvy. Thus, intelligent sensors with a high level of autonomy in their operation are required.

Smart living environments (such as smart homes) have been proposed as spaces instrumented with sensors and actuators to provide personalized, anticipatory, and adaptive services in many areas such as energy management, healthcare, quality of life (independent and assisted living) or social isolation [22,23]. However popular the concept of smart homes is, there is not a universally accepted definition for it [24]. However, as Popescu, Rusu, Bacali, and Popescu explain, Acampora, Cook, Rashidi, and Vasilakos [25] identified the following descriptive features that are very helpful to label any given environment as a smart home or not:

- Context aware: exploiting the contextual information.
- Personalized: to the individual needs.
- Anticipatory: anticipating the individual needs without a conscious intervention.
- Adaptive: to the changing needs.
- Ubiquitous: integrated into the everyday environment.
- Transparency: embedded in an unobtrusive way in the daily life. [24] (p. 115).

Since using novel technologies is not a trivial issue for older adults, ubiquity and transparency features are particularly significant in healthcare monitoring applications, such as the assessment of older adults’ frailty. Transparency is related to the types of activities the elders are requested to carry out while the sensor makes a measurement. A device is considered to comply with transparency requirements when it operates in the background, collecting data without interfering with the elders’ activities of daily living. On the other hand, ubiquity is related to the ability of the device to become part of the context and go unnoticed, just like wall sockets or network routers at home. Together, transparency and ubiquity are also known as unobtrusiveness.

Several models have been proposed to explain frailty, and all of them identify physical performance as a strong frailty marker [3,13]. Therefore, frailty monitoring has usually been based on monitoring phenomena related to mobility, motor skills, and behavior. Examples of the phenomena monitored in usual clinical practice include gait [26] and sit–stand–sit (STS) transitions [27]. The former is widely used to assess slowness; there are several variations of walking tests based on measuring how long it takes a subject to walk along a straight line of different standardized lengths, for example the 4 m walking test (4mWT) and the 10mWT [28]. The complementary approach involves measuring the distance a subject is able to walk during a particular standardized amount of time, for example the 6 min walking test (6minWT) [29]. Usual tests involving STS transitions are used to assess weakness. The 30 s chair-stand test (30s CST), for example, is based on measuring how many sit-to-stand (SiSt) transitions a subject is able to execute during 30 s [30]. The complementary approach is implemented in the five repetitions sit-to-stand test (STS5). This test is based on measuring how long it takes a subject to execute five SiSt transitions [21]. There already are instrumented versions of these tests using sensors to quantify their standard outcomes and even more advanced parameters [31]. However, even though walking and standing up from a chair are usual activities of daily living, the constraints imposed by these kinds of tests require the subjects to interrupt their daily activities to take a measurement. Thus, their instrumented versions do not qualify as transparent activities. The same applies to the instrumented versions of other usual clinical tests such as the Timed Up and Go (TUG) test for physical performance. Even though the different parts of the TUG test are usual daily activities (stand up, walk, turn, and sit down), the specific constraints of the tests prevent them from qualifying as transparent activities. However, on the other hand, there already are commercially available technologies to monitor mobility and motor skills, even in the wild. Smartphones come equipped with GPS receivers able to track people’s location and speed with high reliability [32]. They only work outdoors, but there are pedometers and smart wrist-bands able to count steps and estimate activity levels everywhere [33,34,35]. There are also motion capture systems based on body-worn IMUs able to collect data for detailed kinematic studies [36]. These systems are all examples of body-worn sensors. Not every body-worn sensor has the ability to go unnoticed as required to comply with ubiquity. Since we have not found any lists of objective criteria to assess sensors’ ubiquity, we have applied the following rules: (i) ubiquitous wearable devices are those seamlessly embedded or attached to people’s regular clothing so the monitored person can comfortably wear them for long periods of time; (ii) we use the term ‘on-body sensors’ for those attached to unusual body parts or by unusual means that might make them not comfortable enough to wear them for long periods of time or that might be too apparent and even become a source of stigma; and finally, (iii) non-ubiquitous wearable sensors are those demanding a time-consuming set-up or that are unsuitable for long-term use. Besides body-worn sensors, ambient sensors are those installed or embedded in daily objects that the user does not wear. All ambient sensors are potentially ubiquitous as long as they are not too bulky. Within the present review, we want to identify which of these and other sensing strategies have been observed to provide meaningful information for frailty monitoring applications.

Unobtrusive sensors are expected to work in the background and require minimal set-up, minimal calibration, and minimal maintenance. They are expected to work without the intervention of any qualified personnel over a long time. In order to do so, they need additional information about the actual context. This is a big difference compared to the controlled conditions of a laboratory setting. In the lab, the context of transparent activities can be simulated under controlled conditions. For example, having a wearable sensor to analyze the kinematics of a SiSt transition in the lab does not require the sensor to run an algorithm for the automatic detection of the transition. The research team can manually start and stop the measurement or manually delimit the beginning and the end of the signal. In contrast, such an algorithm is indeed necessary in unsupervised conditions. Similarly, having a wearable sensor to analyze gait patterns in the wild requires the sensor to run an algorithm to identify walking bouts and to select those eligible for further analysis. Additionally, the values of some functional variables in the wild might have a different clinical meaning than the values obtained with a usual clinical test. Therefore, devices tested in unsupervised conditions involve additional and more complex levels of technical development and experimental research. Within the present review, we want to identify which devices have reached that level of development.

Sensors provide an outcome parameter. The value of this parameter quantifies some characteristic feature of the specific phenomenon the sensor is measuring. For example, different parameters can be defined to describe gait, such as gait speed, gait variability, step regularity, gait symmetry, and complexity [37]. On the other hand, a given functional variable may be operationalized in different ways. For example, weakness may be represented by the time spent in a STS5 test [21], or by the number of SiSt transitions in a 30 s chair-stand test (30-s CST) [30]. Then, a relationship between the outcome parameter and a specific operationalization of the target functional variable has to be established. Sensors are expected to provide an estimation for the value of the target functional variables. The value of some functional variables is related to a measurable magnitude that can be directly estimated by the outcome parameter of the sensor. That is the case of the estimation of gait speed from the readings of an accelerometer [38]. In these cases, it is possible to obtain a measurement of the error or accuracy of the sensors’ measurements. On the other hand, the outcomes of some sensors are not a direct estimation of the value of the target functional variable. For example, frailty level is not related to a measurable magnitude. In these cases, the relationship between the outcome parameter of the sensor and the target functional variable can be assessed by a test of statistical association. For example, a *t*-test or an ANOVA test with frailty level as the independent variable can be conducted to test its association with the number of high-activity bouts within a day. Statistical tests of association, however, do not provide an estimation for the value of the target variable. Fortunately, it is still possible to transform the value of the outcome parameter into an estimation for the target functional variable by training a regression or a machine learning model. Then, the quality of the resulting estimation can be objectively assessed, for example, with an area under the curve (AUC) analysis. We undertook the present scoping review to map the literature on sensor-based unobtrusive monitoring of older adults’ frailty by addressing the following research questions:

- RQ1: What types of devices comply with transparency and ubiquity requirements?
- RQ2: Which functional variables have been assessed under transparent and ubiquitous conditions?
- RQ3: Which devices have been tested in unsupervised conditions?
- RQ4: How do the sensor outcomes relate to the target functional variables?
- RQ5: Which functional variables have been assessed with each transparent and ubiquitous or on-body sensor?

There are some previous systematic reviews on related topics. The closest reviews we have found were conducted by Mugueta-Aguinaga and Garcia-Zapirain (2017) [39] and Dasenbrock et al. (2016) [40]. Both reviewed technologies involved in the diagnosis, screening, and monitoring of frailty (the first one also included technologies for treatment, care, and fall prevention [39]). These reviews focus on identifying which sensors are able to identify different frailty levels (i.e., robust, pre-frail, frail). The present review goes beyond that scope in a twofold manner. On the one hand, the present review aims to identify which sensing approaches comply with ubiquity and transparency requirements and are suitable to be used in unsupervised conditions. On the other hand, it is not restricted to technologies assessing frailty level, but includes technologies assessing additional functional variables such as the individual variables involved in the Fried’s phenotype. Additionally, the most recent articles included in these previous reviews were published around five years ago. The first review covered the period 2005–2015 [39], and the second one included papers as recent as 2016 [40]. As explained in the results section, these reviews coincided with a remarkable increase in the number of articles per year in the field between 2014 and 2016, compared to previous years. Similarly, there has been another remarkable increase starting in 2017; therefore, there is a remarkable amount of available novel articles that were not covered by these previous reviews. There is another recent systematic review by Jonkman et al. (2018) on the assessment of physical activity [41]. It was restricted to studies including an evaluation of an intervention that aimed to promote physical activity and/or reduce sedentary behavior. Additionally, the interventions were aimed at the older general population without a focus on the frailty domain. The most recent papers included in other systematic reviews focusing on gait speed [42], kinematic parameters of sit-to-stand and stand-to-sit movements [31], and physical activity [43] were published over seven years ago.

In our present review, we observed that most of the types of sensors that can be worn on the body are not really seamlessly integrated into regular garments and require ad-hoc placement. Only inertial measurement units (IMUs) on the waist (e.g., attached to a regular belt) and wrist-worn sensors are wearable devices complying with ubiquity requirements. On the other hand, all three types of transparent ambient sensors (embedded binary sensors, Kinect^®^ sensors, and beacons) report presence. We also observed that weakness, slowness, exhaustion, and physical activity variables of the Fried’s phenotype, as well as the frailty level itself, have been unobtrusively assessed with an IMU on the waist. However, in unsupervised conditions, it has only provided value estimations for slowness and physical activity. On the other hand, ambient sensors in unsupervised conditions have provided value estimations or predictions for frailty as defined by the Fried’s phenotype (beacons), slowness and performance (embedded binary sensors), and physical activity (Kinect^®^).

## 2. Materials and Methods

The present study was conducted as a scoping review, according to the recommendations of the Joanna Briggs Institute (JBI) as reported in the JBI’s Reviewers Manual [44]. The results have been reported according to the recommendations of the JBI [44] and the PRISMA extension for scoping reviews (PRIMSA-ScR) [45]. Prior to the design of the present review, SCOPUS, Web of Science, PubMed, and the Cochrane Library were examined to identify the existence of any previously published or currently underway systematic or scoping reviews on a similar or identical topic. We did not locate any reviews describing the unobtrusiveness of sensors for the assessment of older adults’ frailty.

### 2.1. Eligibility Criteria

The eligibility criteria were defined using the Population, Concept, Context (PCC) framework, as described in the JBI’s Reviewers Manual [44]. The population of interest for this review is older adults, 65 years old or older. Studies involving either robust, pre-frail, or frail participants were included in the review. Studies involving hospitalized patients were excluded from the review. Studies have been included whether they describe sensors in the form of individual devices or in the form of complex systems comprising multiple hardware or software components. Studies have been included if their sensors were used to assess any of the variables in the following categories: (a) frailty level, (b) any of the variables in the Fried’s phenotype, and (c) usual measurements of performance. Studies have not been included if they measured functional decline due to specific symptoms of a particular pathology, in particular, Parkinson’s disease (PD), falls, and cognitive issues. Studies conducted in either laboratory settings or the usual dwellings of the participants were included in the review.

All kinds of experimental and observational studies have been considered. Only studies published in English language were included. Articles in scientific journals, contributions to conferences, and book chapters were included in this review. Reviews or unpublished and gray literature were not included in this review.

### 2.2. Information Sources and Search Strategy

We conducted a three-step search approach as recommended in the JBI’s Reviewers Manual [44]. The first step consisted of an initial limited search on MEDLINE (PubMed) and SCOPUS. This search was based on an initial search strategy, drafted by the first author and presented in Appendix A. The titles and abstracts in the search results were analyzed for relevant keywords in the topic. The index terms used to describe the articles were retrieved as well. A second version of the search strategy was drafted by the first author by including the identified keywords and index terms and further refined through team discussion. The final search strategies can be found in Appendix B. In the second step, MEDLINE (PubMed), SCOPUS, and Web of Science, as in Millor et al. (2014) [31], were searched for English-language documents published between 2010 and December 2020. The search results were exported into Zotero, and duplicates were removed by the first author. Finally, the third step consisted of an exploration of the reference list of the studies included for qualitative analysis. 

### 2.3. Selection of Studies

Four reviewers (A.C., E.V.-M., X.F., and R.P.-R.) worked in pairs to screen the titles and abstracts according to the eligibility criteria in Section 2.1. In the case of disagreement within any of the pairs, all five members of the team met to discuss and decide. The same four reviewers worked again in pairs to assess full texts for eligibility. Again, in case of disagreement within any of the pairs, all five members of the team met to discuss and decide.

### 2.4. Data Charting

A data-charting form in LibreOffice Calc v6.4.6.2 was jointly developed by the team to determine which variables to extract. After pilot-testing the charting form on five of the papers included for analysis, two reviewers processed each article. The charting form included both descriptive and narrative variables. Descriptive variables comprised author, publication year, functional variable under assessment, phenomenon quantified by the sensor, outcome parameter of the sensor, type of device or system, type of data collection activities, type of relationship between the functional variable and the sensor outcome, and method used to assess said relationship. Some examples of functional variables are frailty level or any of the functional variables in the Fried’s phenotype and usual measurements of performance (TUG, SPPB, and functional questionnaires). Some examples of phenomena include, among others, sit-to-stand transitions, gait, and activity patterns. Finally, examples of sensor outcomes parameters for gait include speed or stride variability. Narrative variables included data processing method, description of the sample of participants, data collection procedure, and results. 

### 2.5. Synthesis of the Results

A descriptive summary of each study can be found in Table A1 (Appendix C) comprising the following descriptive elements: author and year, functional variable under assessment, phenomenon quantified by the sensor, type of data collection activities, type of device or system, and type of relationship between the target functional variable and the outcome parameter of the sensor.

In order to report compliance with transparency and ubiquity requirements, the studies were first grouped by the phenomenon quantified by the sensor and their corresponding transparency level; then, the studies were grouped by type of sensor and the transparency level and ubiquity of the data collection activities. Second, to report the unobtrusiveness level of the solutions assessing different functional variables, the studies were grouped by functional variable under assessment, ubiquity level of the devices, and transparency level of the data collection activities. Third, to report appropriate devices for unsupervised use, the studies complying with transparency requirements were grouped by type of device, phenomenon quantified by the sensor, and experimental setting. Fourth, to report the type of relationship between the target variable and the outcome parameter of the sensor, the studies were grouped by phenomenon quantified by the sensor, variable under assessment, and type of relationship, first for studies complying with transparency requirements and then for studies not complying with transparency requirements. Finally, to report the ability of different devices to assess multiple functional variables, the studies complying with transparency requirements were grouped by type of device and functional variable.

## 3. Results

**Figure 1:**
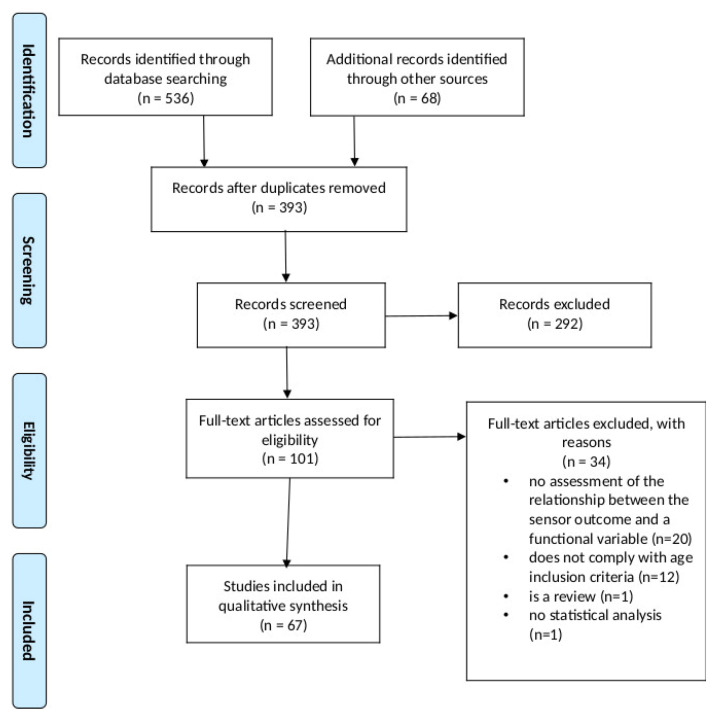
PRISMA flow diagram.

The PRISMA flow diagram in Figure 1 summarizes the outcomes of the different stages in the reviewing process.

**Figure 2:**
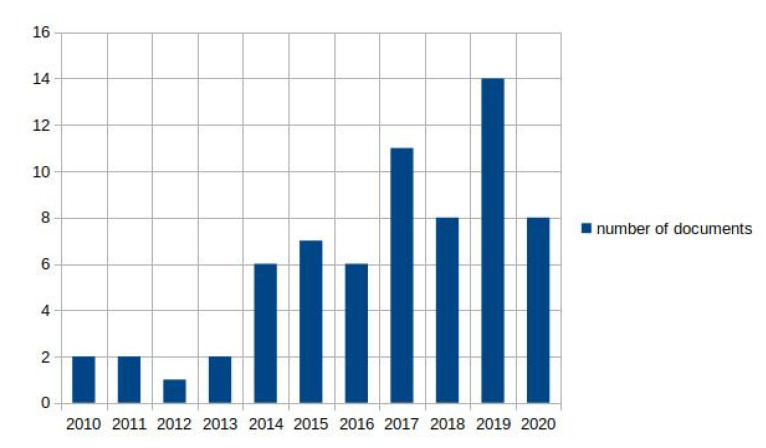
Number of documents over time.

In total, 536 citations were identified from the electronic databases (MEDLINE (PubMed), SCOPUS, and Web of Science), and 68 additional citations were identified through other sources. Forty-three of them came from citations previously identified by the review team, and 25 of them came from scanning the list of reference of the studies included for analysis in subsequent stages of the review. Out of these, 393 citations entered the screening stage after removing duplicates, and 292 citations were excluded during the screening stage based on the information in the documents’ titles and abstracts. The full texts of the remaining 101 citations were retrieved and assessed for eligibility. Of these, 34 documents were excluded for the following reasons: 20 of them did not assess the relationship between the sensor outcome and any functional variables, 12 of them did not comply with the age inclusion criteria, one of them was a review, and another one involved a single participant, and their results were not based on any statistical analyses. Thus, 67 studies were included for analysis in this review. Of these, 59 documents (88%) were journal articles, six documents (9%) were contributions to conferences, and two documents (3%) were book chapters. The number of documents over time is depicted in Figure 2. There is a trend to an annual increase in the number of publications from 2014 onwards, with a notable exception corresponding to the year 2020.

A descriptive summary of each study can be found in Table A1 (Appendix C). The data reported in the following subsections address the different research questions defined in Section 1.

### 3.1. RQ1: What Types of Devices Comply with Transparency and Ubiquity Requirements?

Table 1 lists the different phenomena that have been quantified by the sensors included in this review together with the number of studies complying with transparency requirements for each phenomenon. 

Table 2 lists the different types of devices that have been described in the studies included in this review together with their level of ubiquity and the number of studies where they have been used in transparency conditions.

In Table 3, we have aggregated the studies in each type of ubiquity level (i.e., ubiquitous, on-body, and non-ubiquitous), keeping the difference between transparent and non-transparent activities. 

### 3.2. RQ2: Which Functional Variables Have Been Assessed under Transparent and Ubiquitous Conditions?

Table 4 shows the number of sensor-based studies assessing frailty level. The figures are displayed according to the sensors’ ubiquity and transparency levels.

Table 5 shows the number of sensor-based studies assessing each of the Fried’s variables. The figures are displayed according to the sensors’ ubiquity and transparency levels.

Table 6 shows the number of sensor-based studies assessing physical performance. Once again, the figures are displayed according to the sensors’ ubiquity and transparency levels.

Finally, Table 7 shows the number of studies assessing sensors’ responsiveness according to the sensors’ ubiquity and transparency levels. 

Percentages across all four tables do not add to 100%, because several papers have measured several functional variables with different ubiquity and transparency levels.

### 3.3. RQ3: Which Devices Have Been Tested in Unsupervised Conditions?

Table 8 shows the number of transparent studies and the number of unsupervised studies for each sensor and phenomenon.

### 3.4. RQ4: How do the Sensor Outcomes Relate to the Target Functional Variables?

Table 9 shows the number of studies using a direct or an indirect approach for each phenomenon and functional variable for studies relying on transparent activities.

Table 10 shows the number of studies using a direct or an indirect approach for each phenomenon and functional variable for studies relying on non-transparent activities. 

### 3.5. RQ5: Which Functional Variables Have Been Assessed with Each Transparent and Ubiquitous or On-Body Sensor?

Thus, Table 11 shows the list of functional variables that have been transparently assessed with ubiquitous or on-body sensors. 

## 4. Discussion

The present paper reports the results of a scoping review to map the literature on sensor-based unobtrusive monitoring of older adults’ frailty for the prevention of disability. We observed that most of the types of sensors that can be worn on the body require ad-hoc placement; they are not really seamlessly integrated into regular garments; therefore, they do not go unnoticed. Only IMUs on the waist (e.g., attached to a regular belt) and wrist-worn sensors are wearable devices complying with ubiquity requirements. IMUs on the waist in particular have been used to unobtrusively assess frailty level as well as most of the criteria in Fried’s phenotype (weakness, slowness, exhaustion, and physical activity). Not all these unobtrusive studies based on an IMU on the waist have been conducted in unsupervised conditions; only studies assessing frailty level, slowness, exhaustion, and physical activities have. Moreover, the outcomes of the sensors in these unsupervised studies have provided value estimations just for slowness and physical activity; in the case of frailty level and exhaustion, only statistical associations between the sensors’ outcome parameter and the functional variable have been assessed.

In the case of ambient sensors, all the sensors described in the selected studies are ubiquitous, but only sensors reporting presence in a room have proven to work transparently. All three types of these transparent ambient sensors (embedded binary sensors, Kinect^®^ sensors, and beacons) have been tested in unsupervised conditions, where they have provided value estimations or predictions for frailty level as defined by the Fried’s phenotype (beacons), slowness and performance (embedded binary sensors), and physical activity (Kinect ^®^).

### 4.1. RQ1: What Types of Devices Comply with Transparency and Ubiquity Requirements?

The results show that devices requiring non-transparent activities to collect data have been described as many times as those enabled to work transparently. Non-ubiquitous devices have barely been considered compared to the devices in either of the remaining ubiquity categories. On the other hand, on-body technologies have been as popular as entirely ubiquitous technologies under transparent operation and more popular than ubiquitous sensors in non-transparent activities. 

The three most studied phenomena are STS transitions (37%), gait patterns (31%), and activity patterns (22%). These phenomena have been measured transparently in many studies (16%, 9%, and 22%, respectively), and so have been mobility patterns (4%), stair climbing (3%), heart rate (1%), and arm movements (1%). All the studies relying on activity patterns (15 studies), mobility patterns (three studies), stair climbing (two studies), heart rate (one study), and arm movements (one study) involved transparent activities and did not involve any non-transparent activities [64,106,107,108,109,110,111]. Together, they represent 29% of all the studies. These groups of studies show a strong imbalance in favor of studies on activity patterns with the rest of the phenomena having a marginal presence. Even though studies on STS transitions and gait patterns are the most numerous, most of them do not monitor transparent activities: 14 out of 25 STS studies [57,58,59,60,61,62,63,64,65,66,67,68,69,70] and 17 out of 21 gait studies [57,58,63,64,70,72,73,77,78,79,80,81,82,83,84,85,86] rely on non-transparent activities.

The vast majority (all but one) of the studies relying on non-transparent activities involve an instrumented version of a standardized clinical test. The five repetitions chair-stand test (STS5) [57,59,60,62,63,64,65,66] and the 30-s CST [58,61,67,68,69,70] in STS studies; the 3 m Walking Test (3-mWT), 4-mWT, 6-minWT, etc. in gait studies [57,58,63,64,70,72,73,77,78,79,80,81,82,83,84,85,86]; the TUG test in up-and-go studies [59,62,64,99,100,101,102,103]; the SPPB balance test [57,64], quiet standing test [63], one-foot eyes-closed [58,70] Romberg’s test [104,105], or two-feet eyes-closed [86] in balance studies; a weight scale in weight studies [58,70,77]; the SF-36 and mini-GDS [70,77] in studies based on digitized questionnaires; the Jamar dynamometer in grip studies [77]; and the counter movement jump test in leg extension studies [64]. Only the study based on repetitive elbow flexion did not involve a standardized clinical test [112].

In over half of the studies relying on transparent activities (23 out of 37—please note that [71,73] are include in both activity and gait patterns), the participants were monitored while carrying out their daily activities at home over several days: 14 out of 15 studies on activity patterns [71,73,86,87,88,89,90,91,92,93,94,95,96,97]; all six studies on gait patterns [71,72,73,74,75,76]; all three studies on mobility patterns [106,107,108]; and two studies on STS transitions [55,56]. However, the remaining 14 studies monitored the participants during simulated activities in the lab. Nine studies on STS transitions replicated situations where people stand up and sit back down after performing some additional tasks by analyzing elders’ movements during a single SiSt or stand-to-sit (StSi) transition [46,47,48,49,50,51,52,53,54]. One of the studies on activity level [98] and the study on heart rate [110] monitored the participants while they traveled across a circuit of different daily activities in the lab. The sensors in both studies on stair climbing were tested in controlled conditions without an algorithm for the automatic detection of stairs [64,109]. Finally, arm movements were monitored during the execution of a predefined procedure while buying a 1 kg package of salt in a supermarket [111].

Transparent studies relying on STS transitions measured parameters such as duration, acceleration (mean and maximal), velocity (mean and maximal), trunk angular velocity (mean and maximal), maximal jerk, peak power, vertical acceleration range, and number of postural transitions [46,47,48,49,50,51,52,53,54,55,56]. Transparent studies on gait patterns measured parameters such as gait speed, gait variability, gait asymmetry, gait irregularity, daily walking time, total walking duration, and number of walks per day [71,72,73,74,75,76]. Studies on activity patterns measured parameters such as sedentary behavior, time spent active, and step counts [71,73,86,87,88,89,90,91,92,93,94,95,96,97,98]. Studies on mobility measured parameters such as the number of transitions between different rooms, the time spent in each room, and some other features derived from them [106,107,108]. Studies on stair climbing measured average power and peak power [64,109]. The study on heart rate measured heart rate variability quantified with the triangular index [110]. Finally, the study on arm movements measured mean, standard deviation, skewness, kurtosis, maximum, minimum, amplitude, and energy of 3D acceleration, 3D gyroscope, and heart rate [111].

The three most studied devices are IMUs on the chest (24%), IMUs on the lower back (21%), and IMUs on the waist (15%). All three of them are body-worn sensors. However, only waist-mounted IMUs are ubiquitous wearable sensors. Precisely, only ubiquitous wearable sensors among all body-worn devices have involved transparent activities in all the studies: 10 studies with waist-mounted IMUs [47,53,73,74,87,89,90,94,95,96] and two studies with wrist-worn sensors [110,111]. Similarly to waist-mounted IMUs, nine studies involving an IMU on the chest have involved transparent activities [46,47,48,49,55,56,71,86,97] (which only represent around half of all the studies with a chest-mounted IMU). However, while only two of the 10 studies with waist-mounted IMUs used simulated transparent activities, five of the nine studies on chest-mounted IMUs did. Finally, only five studies involving an IMU on the lower back involved transparent activities [50,51,52,72,109], which represent around a third of all studies with a lower-back-mounted IMU (five out of 14). Some ubiquitous ambient sensors have been used only for non-transparent activities, in particular, distance sensors on furniture/walls [57,62,67,77], load sensors in furniture [57,62,64,77], pressure sensors in furniture [58,70,77,83], and an app in a tablet [77]. In contrast, transparent activities were present when the ubiquitous ambient sensors did not require the subject to interact with any specific objects. This is the case of some binary sensors on the walls [64,76,108], RF beacons [106,107], and the Kinect^®^ sensor [93]. 

Ubiquitous ambient sensors embedded in different pieces of furniture or daily objects include load sensors in a chair [57,58,62,64,70], distance sensors in a chair (either laser-based [57,62] or ultrasound-based [67]), a Doppler sensor in an ornament [77], a bathroom scale [77], pressure sensors in a grip ball [77], pressure sensors on the tip of a cane [83], and a mobile app in a tablet [77]. On the other hand, ubiquitous ambient sensors on the walls, the ceiling, or the floor include laser-based distance sensors [57], Bluetooth beacons for localization [106,107], a Kinect^®^ sensor [93,99,101], passive infra-red sensors as presence detectors [76,108], light barriers [64], ultrasound sensors [81,85] as binary triggers, and load and pressure sensors [57,58,70]). 

### 4.2. RQ2: Which Functional Variables Have Been Assessed under Transparent and Ubiquitous Conditions?

We have found studies for all three categories of functional variables (i.e., frailty level, Fried’s variables, and performance). Additionally, we found a fourth group of studies focused on assessing the responsiveness of the sensor outcomes to the effects of exercise interventions. We found that all the Fried’s criteria (i.e., slowness, weakness, exhaustion, weight loss, and physical activity) have sometimes been the object of assessment in sensor-based studies. Nevertheless, ‘weight loss’ has been assessed only in two studies (3%), and it is the only variable that has not been studied in unobtrusive conditions in any of the studies. Exhaustion has barely been assessed with sensors as well, with only three studies (4%). Most of the studies have focused on the assessment of frailty level, slowness, weakness, and performance. The most common combinations across all four groups of studies are on-body and ubiquitous transparent measurement of frailty levels (13 and 10%, respectively), ubiquitous non-transparent measurement of slowness and weakness (12 and 10%, respectively), and ubiquitous transparent and non-transparent measurement of performance (9%). Studies assessing frailty level with transparent activities (17 studies) add to 60% of all studies in the frailty-level category. In the case of studies assessing performance, the sum decreases to 45% (10 studies). In the case of studies assessing slowness and weakness, the figure goes down to 37% (seven studies) and 33% (eight studies), respectively. In all cases, the studies are equally divided between ubiquitous and on-body sensors.

The most common definition of frailty has been Fried’s phenotype [48,49,56,68,70,71,75,77,79,86,88,94,102,103,104,105,106,107,111,112]. However, a few studies have used different frailty scales such as Rockwood’s Frailty Index and number of disabilities [46,83], Groningen Frailty Indicator [47], Frailty Trait Scale [89,90], and Tillbur Frailty Indicator (TFI) [101]. Frailty level has been operationalized using three levels (e.g., robust, pre-frail, and frail) in 10 studies [68,70,77,79,86,104,106,107,111,112]. A similar number of studies (13 studies) have used only two levels (e.g., robust and pre-frail/frail) [46,47,48,49,56,71,75,94,101,102,103,105,106]. Finally, four studies have used a continuous scale of measurement [83,88,89,90]. Slowness has been operationalized as speed of gait [51,52,63,73,74,76,83,85]. Different studies have operationalized weakness as the stopwatch measurement in an STS5 test [47,59,60,62], the number of SiSt transitions in a 30-s CST [51,58,61,67], lower limb muscle power [54,55,63,78], and grip strength [50,63,77]. The original Fried scale measured weakness of upper limbs, not lower limbs. Exhaustion has been operationalized as the outcome of a questionnaire, either the Pittsburgh Fatigability Scale (PFS) [73] or the Mini-GDS questionnaire [77]. Weight has been operationalized as the measurement of a weight scale [77]. Physical activity has been operationalized as an activity profile (i.e., sedentary vs. active) [77,82], step count [98], time active [65,93,95], and as the outcome of a questionnaire [63,96]. The performance category in this study comprises different assessment methods such as the TUG test [47,55,58,59,62,99,100,110], the SPPB test [57,63,73,97,110], and some questionnaires such as the ADL questionnaire [108], the SF-36 [65,87,92,96,110], and the senior fitness test (SFT) [91]. Among the studies assessing responsiveness, three studies assessed the changes in the sensor outcomes before and after exercise interventions [49,53,69], and one study assessed test–retest reproducibility of the measurements in the absence of intervention [66]. 

### 4.3. RQ3: Which Devices Have Been Tested in Unsupervised Conditions?

Not all the ubiquitous wearable sensors have been used in unsupervised conditions. Only IMUs on the waist have. They have mostly been used to quantify activity patterns (seven studies) and, to a significant lesser degree, to quantify gait patterns (two studies). In contrast, none of the wrist-worn sensors (either heart rate monitors or IMUs) have been used in unsupervised conditions; they have only been used in two different studies involving simulated transparent activities. In the case of on-body sensors, all of them have been used in unsupervised conditions. IMUs on the chest have been used to quantify STS transitions (two studies), gait patterns (one study), and activity patterns (two studies); IMUs on the lower back have been used to quantify gait (one study) and stair climbing (one study); IMUs on the arm have been used to quantify activity patterns (one study); IMUs on the thigh have been used to quantify STS transitions (one study); and even a couple of studies not reporting the IMUs locations have been used to quantify gait (one study) and activity patterns (one study). Finally, only those ubiquitous ambient sensors configured to detect the presence of a subject in a room have been used in unsupervised conditions. Embedded binary sensors, in particular, passive infrared motion sensors, have been used to quantify gait (one study) and mobility patterns (one study); a Kinect^®^ sensor has been used to quantify activity patterns (one study); and Bluetooth beacons in combination with a portable Bluetooth receiver have been used to quantify mobility patterns (two studies). In contrast, ambient sensors configured to measure magnitudes different from presence were not used in unsupervised studies. This is the case of distance sensors (either in furniture or on the walls), load and pressure sensors in the furniture, and mobile applications. Regardless, the unsupervised use of a sensor is not synonymous with unobtrusiveness. For example, the unsupervised IMU on the thigh in [61] was used to assess weakness during a 30-s CST, which is not even a transparent activity. However, the participants in the study were able to take several measurements without any professional help over a month by following a set of digitized instructions in a mobile app.

The most common unsupervised phenomenon is activity patterns (13 studies). Even though they have been studied with sensors in all levels of ubiquity, half of them (seven studies) have been conducted with an IMU on the waist (which is the only ubiquitous wearable device tested in unsupervised conditions). In fact, only one of the eight studies with an unsupervised IMU on the waist does not involve the quantification of activity patterns. We wondered whether that meant an IMU on the waist is restricted to or at least focused on unsupervised assessment of physical activity. In contrast, we found that it has been used on the unsupervised assessment of frailty level [89,90,94], slowness [73,74], performance [73,87], exhaustion [73], and, of course, physical activity [95,96]. 

The vast majority of studies involving transparent activities have been tested in unsupervised conditions regardless of the particular choice of sensor or the target phenomenon. However, studies on transparent STS transitions show a rather different behavior. Even though these are, together with activity patterns, the most common type of transparent studies (11 studies), only two of them have been conducted in unsupervised conditions. The remaining nine studies rely on simulated transparent activities in the lab. This observations suggests that detecting STS transitions in the wild with a single body-worn sensor is still a challenge. 

The length of the experimental stage varies across different types of sensors. Participants are usually requested to wear body-worn sensors (either wearable and on-body) for around seven days, regardless of the selected phenomenon (STS transitions [55], climbing stairs [109], or gait and activity [72,73,74,89,90,91,95,96]). In this last case, the records might be included for analysis if they contain data for five days. Only a few studies required the participants to wear the devices for fewer days (two days) to quantify STS transitions [56] and gait and activity [71,75,88,92]. Only one study required participants to wear their device long term (three weeks) [94]. In contrast, the usual experimental time span for ubiquitous ambient sensors is much longer. Gait or activity patterns used an array of passive infrared sensors on the ceiling for four weeks, close to the participants annual clinical evaluation in one case [76] and a Kinect^®^ sensor for ten months in the other case [93]. Similarly, passive infrared sensors, distributed across the different rooms in the home, were used to quantify mobility patterns for approximately one year [108]. In contrast, the beacon-based system described in [106,107] was used to quantify mobility patterns for only a week.

Body-worn devices report, by definition, data from a single person. In contrast, some ubiquitous ambient sensors, such as ubiquitous PIR motion sensors, have difficulties telling the difference between the sensor readings coming from different dwellers [76,108]. This drawback can be overcome by using cameras such as the Kinect^®^ sensor [93]; however, this approach may raise some privacy concerns. On the other hand, Tegou et al. were able to identify individual mobility patterns by using Bluetooth beacons as ambient sensors and having a smartphone carried by each individual to estimate the user’s location based on the signal received by the smartphone Bluetooth receiver [106,107]. This approach seems to solve the identification problem; however, most people do not carry their phones with them while at home. Additionally, while PIR motion sensors are sensitive to different levels of activity within a room, the beacon-based system cannot tell when the subject is still or on the move unless he goes to a different room.

### 4.4. RQ4: How Do the Sensor Outcomes Relate to the Target Functional Variables?

Most of the studies (seven out of 10) relying on non-transparent STS transitions focus on the assessment of weakness with direct measurements [57,58,59,60,61,62,67]. This observation suggests that STS transitions are a convenient phenomenon to directly assess weakness; however, only two out of 11 studies relying on transparent STS transitions assessed weakness with direct measurements [54,55] with the focus of interest moving towards indirect measurements of frailty level [46,47,48,49,55,56] and including indirect measurements of slowness [51,52], performance [47,55], and even weakness itself [47,50,51]. Analogously, even though the relationship between activity patterns and the level of physical activity seems obvious, there are more studies on indirect measurements of frailty level [71,86,88,89,90,94] and performance [87,91,92,97] than on direct measurements of physical activity [93,95,98]. In the case of studies relying on transparent modalities of gait, the focus is on direct measurements of slowness [72,73,74,76] and indirect measurements of frailty level [71,75], weakness [76], exhaustion [73], and performance [73,76].

The most frequent combinations for transparent activities are indirect measurements of frailty level based on STS transitions or activity (9% each), direct measurements of slowness based on gait (6%), and indirect measurements of performance based on activity patterns (6%). Most transparent and indirect measurements (68%) assess statistical associations [47,48,50,51,52,55,73,75,76,87,88,89,90,91,92,94,96,97,110]. Only 39% estimate or predict a value for the target functional variable [46,47,49,56,71,75,86,106,107,108,111]; all of them but one ([108]) make a prediction about frailty level. This means that further research is still necessary to move indirect measurements of slowness, weakness, performance, exhaustion, and physical activities beyond statistical associations and translate them into value estimations. The sum does not add up to 100%, because two studies describe both statistical associations and value estimations. 

The most frequent combinations for non-transparent activities are direct measurements of weakness based on STS transitions (10%) and direct measurements of slowness based on gait (10%). Among indirect and non-transparent studies, the difference between the percentage of studies based on estimating a value for the target functional variable (50%) [68,79,82,84,86,102,104,112] and on assessing statistical associations (69%) [63,65,78,79,80,83,101,102,103,105,112] is lower than among studies relying on transparent activities. Again, the sum does not add up to 100%, because three studies describe both statistical associations and value estimations.

### 4.5. RQ5: Which Functional Variables Have Been Assessed with Each Transparent and Ubiquitous or On-Body Sensor?

IMUs on the waist are the most versatile type of sensors. They have been used to assess all but one (weight loss) functional variables considered in the present review (i.e., frailty level [47,89,90,94], slowness [73,74], weakness [47], exhaustion [73], physical activity [95,96], performance [47,73,87], and responsiveness [53]). Among those functional variables, only weakness was not assessed in unsupervised conditions [47]. However, this type of sensor has been observed to estimate or predict a value just for two of the five remaining variables, namely, slowness [73,74] and physical activity [95]. Ubiquitous wrist-worn wearables have not gained much attention; only two studies made use of them for the assessment of frailty level in one case [111] and performance in the other case [110]. None of them was even conducted in unsupervised conditions. Analogously, on-body sensors on the arm have been scarcely used (to assess performance [91]). However, other on-body sensors on the trunk, such as IMUs on the chest (frailty level [46,47,48,49,55,56,71,86], weakness [47,55], performance [47,55,97], and responsiveness [49]) and IMUs on the lower back (slowness [51,52,72] and weakness [50,51,109]) have gained more attention. This might mean that researchers have been prioritizing lowering the computational complexity of the algorithms for movement analysis over the ubiquity of the solutions. 

Six studies have used ambient sensors. They involve three different types of technologies (PIR motion sensors, a Kinect^®^ sensor, and Bluetooth beacons), all of them acting as presence detectors. Each type of technology has been used to assess a different set of functional variables: slowness [76,108], weakness [64,76], and performance [76,108] with PIR motion sensors; physical activity [93] with the Kinect^®^ sensor; and frailty level [106,107] with only Bluetooth beacons and a smartphone. Altogether, they are able to assess the same functional variables as the IMU on the waist (exchanging exhaustion for weakness), but they are indeed able to estimate or predict a value for all but one of them (slowness [76,108], performance [108], physical activity [93], and frailty level [106,107]). Since all of these ambient sensors acted as presence detectors, we wonder whether it would be possible to measure all those variables using only one of the technologies. Detecting presence at room level with a Kinect^®^ sensor would require installing one of them in each room, which is rather expensive. In contrast, PIR motion sensors can indeed quantify activity levels within a room by counting the number and frequency of the sensor firings. The beacon-based system cannot quantify activity levels within a room. The beacon-based system provides user location at room level, which is provided by the PIR motion sensors as well. Thus, PIR motion sensors are the most promising ambient sensors to unobtrusively monitor frailty level, slowness, physical activity, and performance in unsupervised conditions.

The focus of the studies varies depending on the functional variable and device as well. The studies assessing frailty level under transparency conditions with a sensor on the chest or a sensor on the waist have focused on searching the outcome parameters better predicting frailty level. The question remains an open topic with great variability across studies. Each study explores a different parameter or set of parameters, even when they rely on the same type of phenomenon (either gait [71,86], STS transitions [46,47,49,55,56], or activity patterns [89,90,94]). Furthermore, comparing the results across studies is hard, because different studies use different metrics to operationalize frailty level: two levels (Fried’s, Rockwood’s, and Groningen frailty scales) [46,47,48,49,56,71,94], three levels (Fried’s scale) [86], and a continuous scale of measurement (Groningen Frailty Index [55] and Frailty Trait Scale [89,90]). 

The studies based on ambient sensors train different machine learning models with feature vectors involving different parameters describing room transitions such as number, duration, or speed, either for assessing frailty level [106,107] or slowness and performance [108]. There is only one study assessing frailty level with a wrist-worn sensor [111]. It is based on training a machine learning model with different time and frequency domain features from raw accelerometer, gyroscope, and heart rate signals [111].

When assessing slowness under transparency conditions with a sensor on the lower back, Czech et al. found that estimating gait speed by continuously monitoring elders for two days resulted in similar gait speed estimations to data from seven to fourteen days [72]. However, even though their sensors have proved to accurately measure the speed of gait in clinical tests in the lab, the measurements collected at home are not very strongly correlated with the clinical measurements [72]. On the other hand, Zarzeczny et al. assessed slowness with a sensor on the lower back not by estimating gait speed, but by using a parameter such as SiSt vertical acceleration range. This parameter showed better correlation with the outcomes of a 6-minWT. In any case, it is still necessary to test whether changes in these parameters can be used as indicators of changes in slowness [51]. 

When assessing slowness under transparency conditions with a sensor on the waist, Mueller et al. observed that compliance patterns among elders were highly variable [74]. Again, even though their sensor proved to accurately measure speed of gait in clinical tests in laboratory environments, the measurements collected at home in unsupervised conditions were consistently lower that the clinical measurements [74]; nevertheless, they observed clinical tests involving longer distances and time duration to be better aligned with measurements of speed in transparent and unsupervised conditions. Urbanek et al. observed that the measurements of other alternative parameters such as acceleration and cadence were lower when measured in free-living conditions than in clinical tests [73].

When assessing slowness under transparency conditions with embedded binary sensors, Kaye et al. used an array of PIR sensors on the ceiling that estimated the participant’s speed every time he walked under the array [76]. Even though they observed a certain association between the sensor estimations and the outcomes of a 9-mWT, the strength of the association was not reported and, once again, the sensor estimations were lower than the measurements from clinical outcomes. On the other hand, Robben et al. reported a low error when predicting the outcomes of a 3-mWT by training a random forest algorithm with the elder’s mobility patterns at home (transitions between rooms, time spent in each room, etc.) [108]. They used a system of around 16 PIR sensors together with some additional ones, distributed across the different rooms in the elder’s home [108].

When assessing weakness under transparency conditions with a sensor on the chest, there have been two different fixation techniques. On the one hand, Zhang et al. used a pendant that showed good test–retest reliability and agreement for peak power, duration, maximal vertical acceleration, and maximum jerk in STS transitions [66]. Subsequently, when they compared the sensor performance in clinical tests, simulated SiSt movements, and free-living conditions, they observed that the measurements of people in the 25th percentile in free-living conditions showed stronger associations with their corresponding clinical measurements than the measurements of people in other percentiles. On the other hand, sensors on the chest have also been fixated with an elastic belt or a harness. Peak power measured by this kind of sensor has shown better correlation with the outcomes of an STS5 than the measurements from a sensor on the waist [47]. 

When assessing weakness under transparency conditions with a sensor on the lower back, two different phenomena have been studied. On the one hand, Hellmers et al. estimated peak and average power from the elders’ movements while climbing stairs [109]. Their sensor combined an IMU and a barometer. They observed low deviations in average power, but the estimations about the height climbed and the time spent did show large deviations [109]. On the other hand, some other works have studied the associations between weakness and some other parameters computed from simulated SiSt transitions. Van Lummel et al. studied associations between hand grip strength and multiple kinematic parameters such as duration, angular range and velocity, and vertical velocity during different phases of the SiSt movement [50]. Zarzeczny et al. studied the correlation between the outcomes of a 30-s CST and the vertical acceleration range from simulated SiSt transitions [51]

When assessing weakness under transparency conditions with binary embedded sensors, Hellmers et al. used light barriers to measure the time spent climbing a stair flight and estimated the average power by a mathematical formula [64]. On the other hand, Kaye et al. used their array of PIR sensors on the ceiling to study the associations between chair-stand outcomes and walks per day, mean speed, variability in walks and speed, etc., but these associations were not significant [76].

All three studies assessing physical activity under transparency conditions rely on monitoring activity, either with a sensor on the waist [95,96] or with a Kinect^®^ ambient sensor [93]. The main focus of these studies was to find a good method and algorithm to classify activities as either active or sedentary; however, their outcome parameters are slightly different from one study to the other (time spent on sedentary behavior [95], time spent active [96], and time spent not sitting [93]). 

### 4.6. Limitations

We conducted our literature search only in three major databases, PubMed, SCOPUS, and Web of Science. Potentially relevant results from other databases have not been included in this review.

We have excluded several studies because their participants did not comply with our age criterion for inclusion. Most of these studies involved people 60 years old or older. After reviewing the abstracts of the excluded studies, we concluded that we have not missed any major approaches. The figures reported in the results, though, might have shown slight differences if these studies had been included.

Many of the studies included in this review applied activities of daily living (ADL) classification technologies to tell the difference between active and sedentary behaviors. The number of included studies on this topic was rather small compared to the large number of studies on ADL classification and activity patterns analysis in the scientific literature, because not all ADL studies in the scientific literature are related to the assessment of functional variables. As a result, we might have missed some studies reporting better classification performances than the included ones. However, those studies better fit in a narrower search focused solely on unobtrusive technologies able to quantify activity patterns.

## 5. Conclusions

We have identified IMUs on the waist as the best candidates to unobtrusively monitor frailty and its related markers in unsupervised conditions. This is because their outcomes have been observed to be related to frailty level itself and to most (all but one) of the variables assessed with the Fried’s criteria, in particular, slowness, weakness, exhaustion, and physical activity. Nevertheless, further research is still necessary to translate the outcomes of these types of sensors into specific predictions of frailty level, weakness, and exhaustion in unsupervised conditions. We have also identified presence detectors as the most promising ambient sensors to unobtrusively monitor frailty level, slowness, physical activity, and performance in unsupervised conditions. Further research could explore the combination of these two technologies.

## Figures and Tables

**Table 1:** Phenomena that have been quantified by the sensors included in the present review (left column) together with the number of studies complying (middle column) and not complying (right column) with transparency requirements. The figures in square brackets are the citations for the corresponding studies. The figures in brackets represent the percentage with respect to all 67 studies in the review.

Phenomenon	Transparent Activities	Non-Transparent Activities
STS transitions (37%)	11 (16%) [46,47,48,49,50,51,52,53,54,55,56]	14 (21%) [57,58,59,60,61,62,63,64,65,66,67,68,69,70]
Gait patterns (31%)	6 (9%) [71,72,73,74,75,76]	17 (25%) [57,58,63,64,70,72,73,77,78,79,80,81,82,83,84,85,86]
Activity patterns (22%)	15 (22%) [71,73,86,87,88,89,90,91,92,93,94,95,96,97,98]	0
Up and go (12%)	0	8 (12%) [59,62,64,99,100,101,102,103]
Balance (12%)	0	8 (12%) [57,58,63,64,70,86,104,105]
Body weight (4%)	0	3 (4%) [58,70,77]
Mobility patterns (4%)	3 (4%) [106,107,108]	0
Stair climbing (3%)	2 (3%) [64,109]	0
Questionnaire (3%)	0	2 (3%) [70,77]
Heart rate (1%)	1 (1%) [110]	0
Arm movements (1%)	1 (1%) [111]	0
Grip patterns (1%)	0	1 (1%) [77]
Elbow flexion (1%)	0	1 (1%) [112]
Leg extension (1%)	0	1 (1%) [64]

**Table 2:** Types of devices that have been described in the studies included in the present review (left column) together with their ubiquity level (second column on the left) and the number of Scheme 67 studies in the review. Note that some studies use multiple types of devices; thus, the sum of the percentages in all the different cells does not add up to one hundred.

Device	Ubiquity	Transparent Activities	Non-Transparent Activities
IMU on chest	On-body	9 (13%) [46,47,48,49,55,56,71,86,97]	7 (10%) [59,66,82,84,102,103,105]
IMU on lower back	On-body	5 (7%) [50,51,52,72,109]	10 (15%) [60,62,63,65,68,69,72,78,79,100]
IMU on waist	Yes (wearable)	10 (15%) [47,53,73,74,87,89,90,94,95,96]	1 (1%) [73]
Binary sensors in furniture/walls	Yes (ambient)	3 (4%) [64,76,108]	4 (6%) [62,64,81,85]
IMUs on multiple body parts	No	2 (3%) [54,88]	5 (7%) [80,86,98,104,112]
Distance sensor on furniture/walls	Yes (ambient)	0	4 (6%) [57,62,67,77]
Load sensors in furniture	Yes (ambient)	0	4 (6%) [57,62,64,77]
Pressure sensors in furniture	Yes (ambient)	0	4 (6%) [58,70,77,83]
Kinect sensor	Yes (ambient)	1 (1%) [93]	2 (3%) [99,101]
Beacons and smartphone	Yes (ambient)	2 (3%) [106,107]	0
Heart rate monitor on wrist	Yes (wearable)	2 (3%) [110,111]	0
IMU unknown location	On-body	2 (3%) [75,92]	0
IMUs on wrist	Yes (wearable)	1 (1%) [111]	0
IMU on arm	On-body	1 (1%) [91]	0
IMU on thigh	On-body	0	1 (1%) [61]
App in tablet	Yes (ambient)	0	1 (1%) [77]

**Table 3:** Number of studies describing devices working in transparent (middle column) and non-transparent (right column) activities for each type of ubiquity level. The figures in brackets represent the percentage of studies compared to the total number of studies.

Ubiquity Level	Transparent Activities	Non-Transparent Activities
Ubiquitous	18 (27%)	13 (19%)
On-body	17 (25%)	18 (27%)
Non-ubiquitous	2 (3%)	5 (7%)

**Table 4:** Number of sensor-based studies assessing frailty level. The figures are displayed according to the sensors’ ubiquity and transparency levels. The figures in square brackets are the citations for the corresponding studies. The figures in brackets represent the percentage with respect to all 67 studies in the review.

Ubiquity Level	Transparent Activities	Non-Transparent Activities
Ubiquitous	7 (10%)	3 (4%)
	[47,89,90,94,106,107,111]	[70,83,101]
On-body	9 (13%)	5 (7%)
	[46,47,48,49,55,56,71,75,86]	[68,79,102,103,105]
Non-ubiquitous	1 (1%)	3 (4%)
	[88]	[86,104,112]

**Table 5:** Number of sensor-based studies assessing each of the Fried’s variables. The figures are displayed according to the sensors’ ubiquity and transparency levels. The figures in square brackets are the citations for the corresponding studies. The figures in brackets represent the percentage with respect to all 67 studies in the review.

Fried’s Variable	Ubiquity Level	Transparent Activity	Non-Transparent Activities
Slowness	Ubiquitous	4 (6%) [73,74,76,108]	8 (12%) [57,58,64,73,77,81,83,85]
	On-body	3 (4%) [51,52,72]	2 (3%) [63,72]
	Non-ubiquitous	0	2 (3%) [80,112]
Weakness	Ubiquitous	3 (4%) [47,64,76]	6 (9%) [57,58,62,64,67,77]
	On-body	5 (7%) [47,50,51,55,109]	6 (9%) [59,60,61,62,63,78]
	Non-ubiquitous	1 (1%) [74]	2 (3%) [80,112]
Exhaustion	Ubiquitous	1 (1%) [73]	3 (4%) [70,73,77]
	On-body	0	0
	Non-ubiquitous	0	0
Weight loss	Ubiquitous	0	2 (3%) [58,77]
	On-body	0	0
	Non-ubiquitous	0	0
Physical activity	Ubiquitous	3 (4%) [93,95,96]	2 (3%) [70,77]
	On-body	0	4 (6%) [63,65,82,84]
	Non-ubiquitous	1 (1%) [99]	0

**Table 6:** Number of sensor-based studies assessing physical performance. The figures are displayed according to the sensors’ ubiquity and transparency levels. The figures in square brackets are the citations for the corresponding studies. The figures in brackets represent the percentage with respect to all 67 studies in the review.

Ubiquity Level	Transparent Activities	Non-Transparent Activities
Ubiquitous	6 (9%)	6 (9%)
	[47,73,76,87,108,110]	[57,58,62,64,73,99]
On-body	5 (7%)	4 (6%)
	[47,55,91,92,97]	[59,63,65,100]
Non-ubiquitous	0	1 (1%)
		[80]

**Table 7:** Number of studies assessing the sensors’ responsiveness according to the sensors’ ubiquity and transparency levels. The figures in square brackets are the citations for the corresponding studies. The figures in brackets represent the percentage with respect to all 67 studies in the review.

Ubiquity Level	Transparent Activities	Non-Transparent Activities
Ubiquitous	1 (1%)	0
	[53]	
On-body	1 (1%)	2 (3%)
	[49]	[66,69]
Non-ubiquitous	0	0

**Table 8:** Number of studies in unsupervised conditions (right column) and number of studies relying on transparent activities (second column on the right) for different types of devices (left column) and phenomena (second column on the left). The figures in square brackets are the citations for the corresponding studies. The figures in brackets represent the percentage with respect to all 67 studies in the review. Note that the studies in the unsupervised column are a subset of the studies in the transparent column. The IMU on the thigh in the last row is the only non-transparent sensor tested in unsupervised conditions.

Device	Phenomenon	Transparent Studies	Unsupervised Studies
IMU on chest	STS transitions	6 (9%) [46,47,48,49,55,56]	2 (3%) [55,56]
	Gait	1 (1%) [71]	1 (1%) [71]
	Activity	3 (4%) [71,86,97]	2 (3%) [71,86]
IMU on lower back	STS transitions	3 (4%) [50,51,52]	0
	Gait	1 (1%) [72]	1 (1%) [72]
	Stair climbing	1 (1%) [109]	1 (1%) [109]
IMU on waist	STS transitions	2 (3%) [47,53]	0
	Gait	2 (3%) [73,74]	2 (3%) [73,74]
	Activity	7 (10%) [73,87,89,90,94,95,96]	7 (10%) [73,87,89,90,94,95,96]
Embedded binary sensors	Gait	1 (1%) [76]	1 (1%) [76]
	Mobility	1 (1%) [108]	1 (1%) [108]
	Stair climbing	1 (1%) [64]	0
IMU on multiple body parts	STS transitions	1 (1%) [54]	0
	Activity	2 (3%) [88,98]	1 (1%) [88]
Kinect^®^ sensor	Activity	1 (1%) [93]	1 (1%) [93]
Beacons and smartphone	Mobility	2 (3%) [106,107]	2 (3%) [106,107]
IMU on unknown location	Gait	1 (1%) [75]	1 (1%) [75]
	Activity	1 (1%) [92]	1 (1%) [92]
Heart rate monitor on wrist	Heart rate	1 (1%) [110]	0
IMU on wrist	Arm movements	1 (1%) [111]	0
IMU on arm	Activity	1 (1%) [91]	1 (1%) [91]
IMU on thigh	STS transitions	0	1 (1%) [61]

**Table 9:** Relationships between sensor outcomes (left column) and the target functional variables (second column on the left) for studies relying on transparent activities. Studies where the outcome parameters act as a direct measurement for the value of the target variable are listed in the second column on the right. Studies where the outcome parameters act as an indirect measurement for the value of the target variable are listed on the right column. The figures in square brackets are the citations for the corresponding studies. The figures in brackets represent the percentage with respect to all 67 studies in the review.

Phenomenon (Transparent)	Variable	Direct Measurements	Indirect Measurements
STS transitions	Frailty level	0	6 (9%) [46] ^1^ [47] ^1^ [48] ^2^ [49] ^1^ [55] ^2^ [56] ^1^
	Slowness	0	2 (3%) [51] ^2^ [52] ^2^
	Weakness	2 (3%) [54] ^a^ [55] ^b^	3 (4%) [47] ^3^ [50] ^2^ [51] ^2^
	Performance	0	2 (3%) [47] ^2^ [55] ^2^
	Responsiveness	2 (3%) [49] ^b^ [53] ^a^	0
Gait	Frailty level	0	2 (3%) [71] ^1^ [75] ^3^
	Slowness	4 (6%) [72] ^b^ [73] ^b^ [74] ^c^ [76] ^b^	0
	Weakness	0	1 (1%) [76] ^2^
	Exhaustion	0	1 (1%) [73] ^2^
	Performance	0	2 (3%) [73] ^2^ [76] ^2^
Activity	Frailty level	0	6 (9%) [71] ^1^ [86] ^1^ [88] ^2^ [89] ^2^ [90] ^2^ [94] ^2^
	Physical activity	3 (4%) [93] ^c^ [95] ^a^ [98] ^a^	1 (1%) [96] ^2^
	Performance	0	4 (6%) [87] ^2^ [91] ^2^ [92] ^2^ [97] ^2^
Mobility	Frailty level	0	2 (3%) [106] ^1^ [107] ^1^
	Slowness	0	1 (1%) [108] ^1^
	Performance	0	1 (1%) [108] ^1^
Stair climbing	Weakness	1 (1%) [109] ^a^	0
Heart rate	Performance	0	1 (1%) [110] ^2^
Arm movements	Frailty level	0	1 (1%) [111] ^1^

^a^ studies assessing agreement with a measurement of error. ^b^ studies assessing agreement with a correlation analysis. ^c^ studies assessing agreement with both a measurement of error and a correlation analysis. ^1^ studies estimating or predicting a value for the target variables. ^2^ studies testing statistical associations. ^3^ studies both estimating a value and testing statistical associations.

**Table 10:** Relationships between sensor outcomes (left column) and the target functional variables (second column on the left) for studies relying on non-transparent activities. Studies where the outcome parameters act as a direct measurement for the value of the target variable are listed in the second column on the right. Studies where the outcome parameters act as an indirect measurement for the value of the target variable are listed on the right column. The figures in square brackets are the citations for the corresponding studies. The figures in brackets represent the percentage with respect to the total all 67 studies in the review.

Phenomenon (Non-Transparent)	Variable	Direct Measurements	Indirect Measurements
STS transitions	Frailty level	0	1 (1%) [68] ^1^
	Weakness	7 (10%) [57] ^a^ [58] ^b^ [59] ^a^ [60] ^b^ [61] ^a^ [62] ^b^ [67] ^a^	0
	Physical activity	0	1 (1%) [65] ^2^
	Performance	0	1 (1%) [65] ^2^
	Responsiveness	2 (3%) [66] ^a^ [69] ^b^	0
Gait	Frailty level	0	3 (4%) [79] ^3^ [83] ^2^ [86] ^1^
	Slowness	7 (10%) [57] ^a^ [58] ^b^ [64] ^b^ [72] ^a^ [77] ^a^ [81] ^a^ [85] ^a^	3 (4%) [63] ^2^ [80] ^2^ [83] ^2^
	Weakness	0	3 (4%) [63] ^2^ [78] ^2^ [80] ^2^
	Physical activity	0	3 (4%) [63] ^2^ [82] ^1^ [84] ^1^
	Performance	0	2 (3%) [63] ^2^ [80] ^2^
Up and go	Frailty level	0	3 (4%) [101] ^2^ [102] ^3^ [103] ^2^
	Performance	4 (6%) [59] ^a^ [62] ^b^ [99] ^a^ [100] ^b^	0
Balance	Frailty level	0	3 (4%) [86] [104] [105] ^1^
	Performance	2 (3%) [57] ^a^ [58] ^b^	0
Body weight	Weight loss	2 (3%) [58] ^b^ [77] ^a^	0
Questionnaire	Exhaustion	1 (1%) [77] ^a^	0
	Physical activity	1 (1%) [77] ^a^	0
Grip patterns	Weakness	1 (1%) [77] ^a^	0
Elbow flexion	Frailty level	0	1 (1%) [112] ^1^
	Slowness	0	1 (1%) [112] ^2^
	Weakness	0	1 (1%) [112] ^2^

^a^ studies assessing agreement with a measurement of error. ^b^ studies assessing agreement with a correlation analysis. ^1^ studies estimating or predicting a value for the target variables. ^2^ studies testing statistical associations. ^3^ studies both estimating a value and testing statistical associations.

**Table 11:** Number of studies (right column) describing transparent assessment of any functional variable with each ubiquitous and on-body sensor. The figures in square brackets are the citations for the corresponding studies. The figures in brackets represent the percentage with respect to all 67 studies in the review.

Device	Functional Variable	Number of Studies
IMU on chest	Frailty level	8 (12%) [46,47,48,49,55,56,71,86]
	Weakness	2 (3%) [47,55]
	Performance	3 (4%) [47,55,97]
	Responsiveness	1 (1%) [49]
IMU on lower back	Slowness	3 (4%) [51,52,72]
	Weakness	3 (4%) [50,51,109]
IMU on waist	Frailty level	4 (6%) [47,89,90,94]
	Slowness	2 (3%) [73,74]
	Weakness	1 (1%) [47]
	Exhaustion	1 (1%) [73]
	Physical activity	2 (3%) [95,96]
	Performance	3 (4%) [47,73,87]
	Responsiveness	1 (1%) [53]
Embedded binary sensors	Slowness	2 (3%) [76,108]
	Weakness	2 (3%) [64,76]
	Performance	2 (3%) [76,108]
Kinect^®^ sensor	Physical activity	1 (1%) [93]
Beacons and smartphone	Frailty level	2 (3%) [106,107]
Heart rate monitor on wrist	Frailty level	1 (1%) [111]
	Performance	1 (1%) [110]
IMU on unknown location	Frailty level	1 (1%) [75]
	Performance	1 (1%) [92]
IMU on wrist	Frailty level	1 (1%) [111]
IMU on arm	Performance	1 (1%) [91]

## Appendix A. Initial Search Strategy for MEDLINE (PubMed)

TITLE-ABS-KEY ((“Older people” OR “older adult” OR elder * OR age *) AND (frailty OR “frailty syndrome” OR “functional status” OR “functional decline”) AND (sensor)).

## Appendix B. Final Search Strategies

### Appendix B.1. Final Search Strategy for SCOPUS

TITLE-ABS-KEY ((“older people” OR “older adult” OR elder OR elderly OR senior) AND (frailty OR “frailty syndrome” OR “functional status” OR “functional decline” OR “physical function” OR “physical performance” OR “physical resilience” OR “geriatric assessment” OR “functional abilities” OR “physical decline” OR “functional changes”) AND (sensor OR wearable OR “smart city” OR “internet of things” OR “smart home” OR “smart living environment”) AND NOT (alzheimer OR parkinson OR falls OR dementia OR “cognitive frailty” OR “cognitive impairment”)) AND (LIMIT-TO (PUBYEAR, 2020) OR LIMIT-TO (PUBYEAR, 2019) OR LIMIT-TO (PUBYEAR, 2018) OR LIMIT-TO (PUBYEAR, 2017) OR LIMIT-TO (PUBYEAR, 2016) OR LIMIT-TO (PUBYEAR, 2015) OR LIMIT-TO (PUBYEAR, 2014) OR LIMIT-TO (PUBYEAR, 2013) OR LIMIT-TO (PUBYEAR, 2012) OR LIMIT-TO (PUBYEAR, 2011) OR LIMIT-TO (PUBYEAR, 2010)) AND (LIMIT-TO (LANGUAGE, “English”)).

### Appendix B.2. Final Search Strategy for Web of Science

(TS = ((“older people” OR “older adult” OR elder OR elderly OR senior) AND (frailty OR “frailty syndrome” OR “functional status” OR “functional decline” OR “physical function” OR “physical performance” OR “physical resilience” OR “geriatric assessment” OR “functional abilities” OR “physical decline” OR “functional changes”) AND (sensor OR wearable OR “smart city” OR “internet of things” OR “smart home” OR “smart living environment”)) NOT TS = (alzheimer OR parkinson OR falls OR dementia OR “cognitive frailty” OR “cognitive impairment”)).

This search strategy was further restricted to documents in English language published from 2010 to 2020.

### Appendix B.3. Final Search Strategy for MEDLINE (PubMed)

((“older people” [Text Word] OR “older adult” [Text Word] OR elder[Text Word] OR elderly[Text Word] OR senior[Text Word]) AND (frailty[Text Word] OR “frailty syndrome” [Text Word] OR “functional status” [Text Word] OR “functional decline” [Text Word] OR “physical function” [Text Word] OR “physical performance” [Text Word] OR “physical resilience” [Text Word] OR “geriatric assessment” [Text Word] OR “functional abilities” [Text Word] OR “physical decline” [Text Word] OR “functional changes” [Text Word]) AND (sensor[Text Word] OR wearable[Text Word] OR “smart city” [Text Word] OR “internet of things” [Text Word] OR “smart home” [Text Word] OR “smart living environment” [Text Word])) NOT (alzheimer [Text Word] OR parkinson [Text Word] OR falls[Text Word] OR dementia[Text Word] OR “cognitive frailty” [Text Word] OR “cognitive impairment” [Text Word]).

This search was further restricted to documents published from 2010 to 2020.

## Appendix C. Descriptive Summary of the Studies

The following abbreviations were used in the descriptive summary of the studies.

- Absolute error (Abs. error).
- Accuracy (Acc.).
- Statistical association (Assoc.).
- Area Under the Curve (AUC).
- Chair stand test (CST).
- Degrees of freedom (DoF).
- Intra-class correlation (ICC).
- Mean absolute error (MAE).
- Physical activity (PA).
- Standard error of measurement (SEM).
- Sensitivity (Sens).
- Specificity (Spec).
- Usability (Usab.).

**Table A1:** Descriptive summary of the studies.

Author (Year)	Functional Variable (Operationalization)	Phenomenon	Activity (Transparent/Unsupervised)	Device or System (Ubiquity)	End-Point
1. Reuter (2020) [87]	Performance (RAND 36 Health Survey physical function subscale)	Activity patterns	Yes/yes	ActiGraph GT3x+ on waist (ubiquitous)	Indirect/Assoc. (regression)
2. Kumar (2020) [71]	Frailty level (Fried’s: robust vs. pre-frail/frail)	Gait patterns & Activity patterns	Yes/yes	PAMSys 3D accelerometer on chest (T-shirt pocket) (on-body)	Indirect/Error (AUC)
3. Higueras-Fresnillo (2020) [88]	Frailty level (Fried’s: continuous z-scores)	Activity patterns	Yes/yes	IDEEA five 2D accelerometers on sternum, thighs, and feet (non-ubiquitous)	Indirect/Assoc. (regression)
4. Garcia-Moreno (2020) [111]	Frailty level (Fried’s: robust vs. pre-frail vs. frail)	Arm movements	Yes/no	Samsung Gear S3 Smartwatch: 3D accelerometer, 3D gyroscope, heart rate (ubiquitous)	Indirect/Error (acc, sens, spec)
5. Fudickar (2020) [62]	Performance (TUG stopwatch)	Up and go	No/no	(a) aTUG system (ubiquitous) (b) IMU on lower back (on-body)	Direct/Assoc. (correlation)
	Weakness (STS5 stopwatch)	STS transitions (cycles)	No/no	(a) aTUG system (ubiquitous) (b) IMU on lower back (on-body)	Direct/Assoc. (correlation)
6. Czech (2020) [72]	Slowness (GAITRite)	Gait patterns	(a) no/no (b) yes/yes	(a) Opal APDM IMU on lower back (on-body) (b) Activinsights GENEActive IMU on lower back (on-body)	(a) Direct/error (ICC, Bland-Altman) (b) Direct/assoc (regression, correlation)
7. Cobo (2020) [61]	Weakness (30-s CST transition count)	STS transitions (cylces)	(a) no/no (b) no/yes	Accelerometer on thigh (on-body)	(a) Direct/error (abs. error) (b) usab.
8. Cobo (2020) [67]	Weakness (30-s CST transition count)	STS transitions (cycles)	No/no	Ultrasound distance sensor on chair (ubiquitous)	Direct/Error (ICC)
9. Tegou (2019) [106]	Frailty level (Fried’s: (a) robust vs. prefrail/frail (b) robust vs. prefrail vs. frail)	Mobility patterns	Yes/yes	BT beacons + smartphone (ubiquitous)	Indirect/Error (accuracy)
10. Mueller (2019) [74]	Slowness (walking test stopwatch and speed)	Gait patterns	(a) yes/no (b) yes/yes	Actibelt RCT2 IMU on waist (ubiquitous)	(a) Direct/error (b) Direct/assoc. (correlation)
11. Misu (2019) [80]	Performance (tandem test stopwatch: impaired vs. normal)	Gait patterns	No/no	IMU on heel and lower trunk (multiple body parts) (non-ubiquitous)	Indirect/Assoc. (regression + *t*-test)
	Slowness (walking test speed)				
	Weakness (STS5 stopwatch: impaired vs. normal)				
12. Lepetit (2019) [46]	Frailty level (Rockwood’s: healthy vs. frail)	STS transitions (transitions)	Yes/no	9DoF IMU on chest with strap (on-body)	Indirect/Error (AUC)
13. Kumar (2019) [75]	Frailty level (Fried’s: robust vs. prefrail/frail)	Gait patterns	Yes/yes	3DoF accelerometer on unknown location (on-body)	(a) Indirect/assoc. (regression) (b) Indirect/error (sens, spec)
14. Kim & Won (2019) [81]	Slowness (walknig test speed)	Gait patterns	No/no	Ultrasound sensors as binary triggers (ubiquitous)	Direct/error
15. Jung (2019) [57]	Slowness (SPPB walking test)	Gait patterns	No/no	LiDAR distance sensor on wall (ubiquitous)	Direct/Error (ICC, kappa agreement)
	Weakness (SPPB STS5)	STS transitions (cycles)	No/no	Load-cell and LiDAR distance sensor on chair (ubiquitous)	
	Performance (SPPB balance test)	Balance	No/no	Load-cell array on floor (ubiquitous)	
16. Hellmers (2019) [109]	Weakness (lower limb peak power)	Stair climbing	(a) yes/no (b) yes/yes	Bosch BMA180 3DoF accelerometer and barometer on lower back (on-body)	(a) Direct/error (b) error (accuracy)
17. Hellmers (2019) [60]	Weakness (STS5 stopwatch)	STS transitions (cycles)	No/no	Bosch BMA180 3DoF (and barometer?) on lower back (on-body)	Direct/association (correlation)
18. Graham (2019) [110]	Performance (SPPB, TUG & SF-36)	Heart rate variability	Yes/no	Empatica E4 wrist-worn PPG (ubiquitous)	Indirect/Assoc. (correlation)
19. Galán-Mercant (2019) [82]	Physical activity (PA profile: sedentary vs. insufficiently active)	Gait patterns	No/no	IPhone 4 IMU on chest (on-body)	Indirect/Error (accuracy)
20. Coni (2019) [63]	Physical activity (PA questionnaire)	Gait patterns & balance & STS transitions (cycles)	No/no	Galaxy SII or SIII smartphone accelerometer on lower back (on-body)	Indirect/Assoc. (regression)
	Performance (SPPB)				
	Weakness (grip strength & lower limb muscle power)				
	Slowness (walking test speed)				
21. Chkeir (2019) [77]	Weight loss (weight scale)	Body weight	No/no	Connected bathroom scale (ubiquitous)	Direct/association (correlations)
	Weakness (grip strength)	Grip patterns	No/no	Grip-ball (ubiquitous)	
	Slowness (walking test speed)	Gait patterns	No/no	Environmental Doppler sensor (ubiquitous)	
	Physical activity (PA profile: sedentary vs. insufficiently active)	Questionnaire	No/no	App in a tablet (ubiquitous)	
	Exhaustion (mini-GDS questionnaire)	Questionnaire	No/no	App in a tablet (ubiquitous)	
	Frailty level (Fried’s: robust vs. prefrail vs. frail)	All above	No/no	All above	
22. Ballesteros (2019) [83]	Frailty level (number of disabilities)	Gait patterns	No/no	Instrumented cane (pressure sensors) (ubiquitous)	Indirect/Assoc.
	Slowness (walking test speed)				
23. van Lummel (2018) [50]	Weakness (grip strength)	STS transitions (transitions)	Yes/no	DynaPort Hybrid IMU on lower back (on-body)	Indirect/Assoc.
24. Urbanek (2018) [73]	Performance (SPPB)	Gait patterns	(a) no/no (b) yes/yes	Actigraph GT3X+ accelerometer on right hip (ubiquitous)	Indirect/Assoc. (regression)
	Slowness (walking test speed)	(a)(b) Gait patterns (b) activity patterns	(a) no/no (b) yes/yes		
	Exhaustion (questionnaire)	Gait patterns	(a) no/no (b) yes/yes		
25. Tsipouras (2018) [107]	Frailty level (Fried’s: robust vs. prefrail vs. frail)	Mobility patterns	Yes/yes	BT beacons + smartphone (ubiquitous)	Indirect/Error (accuracy)
26. Mañas (2018) [89]	Frailty level (Frailty Trait Scale)	Activity patterns	Yes/yes	ActiTrainer accelerometer on hip (ubiquitous)	Indirect/Assoc. (regression)
27. Magistro (2018) [98]	Physical activity (step count)	Activity patterns	Yes/no	ADAMO Care Watch 3DoF accelerometer on each wrist. (non-ubiquitous)	Direct/Error (abs. error)
28. Kampel (2018) [99]	Performance (TUG stopwatch)	Up and go	No/no	Kinect sensor (ubiquitous)	Direct/Error.
29. Hellmers (2018) [100]	Performance (TUG stopwatch)	Up and go	No/no	Bosch BMA180 3DoF (and barometer?) on lower back (on-body)	Direct/association (correlation)
30. Galan-Mercant (2018) [84]	Physical activity (PA profile: sedentary vs. insufficiently active)	Gait patterns	No/no	IPhone 4 IMU on chest (on-body)	Indirect/Error (AUC)
31. Zhang (2017) [55]	Weakness (lower limbs peak power)	STS transitions (transitions)	Yes/yes	Pendant with accelerometer and air pressure sensor on chest (on-body)	Direct/Assoc. (correlation)
	Performance (TUG)				Indirect/Assoc. (correlation)
	Frailty level (Groningen Frailty Indicator)				
32. Zarzeczny (2017) [51]	Weakness (30-s CST transitions count)	STS transistions (transitions)	Yes/no	G-Sensor IMU on lower back (on-body)	Indirect/Assoc. (correlation)
	Slowness (walking test speed)				
33. Robben (2017) [108]	Performance (ADL questionnaire)	Mobility patterns	Yes/yes	Binary environmental sensors (ubiquitous)	Indirect/Error (MAE)
	Slowness (walking test speed?)				
34. Pozo-Cruz (2017) [90]	Frailty level (Frailty Trait Scale)	Activity patterns	Yes/yes	ActiGraph 3DoF accelerometer on left hip (ubiquitous)	Indirect/Assoc. (regression)
35. Parvaneh (2017) [56]	Frailty level (Fried’s: robuts vs. prefrail/frail)	STS transitions (transitions)	Yes/yes	PAMSys 3DoF accelerometer on chest (on-body)	Indirect/Error (odds ratio)
36. Millor (2017) [68]	Frailty level (Fried’s: robust vs. prefrail vs. frail)	STS transitions (cycles)	No/no	MTx Orientation Tracker IMU on lower back (on-body)	Indirect/Error (AUC)
37. Jantunen (2017) [91]	Performance (senior fitness test)	Activity patterns	Yes/yes	SenseWear Pro 3 Armband multisensory on triceps (on-body)	Indirect/Assoc. (regression)
38. Hellmers (2017) [64]	Slowness (Fried’s walking test) (SPPB walking test) (6-minWT)	Gait patterns	No/no	Light barriers (ubiquitous)	Direct/Association (correlation)
	Weakness (a) (stair climb power) (b) (SPPB STS5 test) (c) (Counter Movement Jump)	(a) Stair climbing (b) STS transitions (cycles) (c) leg extension	(a) Yes/no (b) No/no (c) No/no	(a) Light barriers (ubiquitous) (b) aTUG (ubiquitous) (c) force platform (ubiquitous)	Already validated
	Performance (TUG)	Up and go	No/no	aTUG (ubiquitous)	Already validated
	Performance (SPPB balance test)	Balance	No/no	Force plate (ubiquitous)	Already validated
39. Ferre (2017) [85]	Slowness (walking test stopwatch)	Gait patterns	No/no	Ultrasound sensors as binary triggers (ubiquitous)	Direct/Error (mean error)
40. Bogen (2017) [92]	Performance (SF-36)	Activity patterns	Yes/yes	accelerometer on unknown location (on-body)	Indirect/Assoc. (regression)
41. Banerjee (2017) [93]	Physical activity (time active)	Activity patterns	Yes/yes	Microsoft kinect below the ceiling (ubiquitous)	Direct/Error (accuracy) Direct/Assoc. (correlation)
42. Weiss (2016) [52]	Slowness (walking test speed)	STS transitions (transitions)	Yes/no	Inertial 3DoF sensor on lower back (on-body)	Indirect/Assoc. (regression)
43. van Lummel (2016) [65]	Performance (SF-36)	STS transitions (cycles)	No/no	DynaPort IMU on lower back (on-body)	Indirect/Assoc. (Mann-Whitney U-test)
	Physical activity (time active)				
44. Martinikorena (2016) [78]	Weakness (lower limbs muscle power)	Gait patterns	No/no	Mtx XSens Orientation Tracker on lower back (on-body)	Indirect/Assoc. (correlation)
45. Lin (2016) [58]	Slowness (???)	Gait patterns	No/no	Pressure sensors in a chair (ubiquitous)	Direct/Assoc. (correlation)
	Weakness (30-s CST transitions count)	STS transitions (cycles)	No/no	Pressure sensor in a chair (ubiquitous)	
	Weight (weight scale)	Body weight	No/no	Pressure sensor in a chair (ubiquitous)	
	Performance (one-foot closed-eyes stopwatch)	Balance	No/no	Pressure sensor on the floor (ubiquitous)	
46. Gianaria (2016) [101]	Frailty level (Tillburg Frailty Indicator: robust vs. frail)	Up and go	No/no	Microsoft kinect sensor (ubuiquitous)	Indirect/Assoc. (correlation)
47. Chan (2016) [59]	Weakness (STS5 stopwatch)	STS transitions (cycles)	No/no	Samsung Galaxy Note II IMU on chest (on-body)	Direct/Error (ICC, Bland-Altman)
	Performance (TUG stopwatch)	Up and go	No/no		
48. Toosizadeh (2015) [104]	Frailty level (Fried’s: robust vs. prefrail vs. frail)	Balance	No/no	9DoF IMU on lower back and on shin (non-ubiquitous)	Indirect/Error (sens, spec)
49. Toosizadeh (2015) [112]	Frailty level (Fried’s: robust vs. prefrail vs. frail)	Elbow flexion	No/no	3DoF gyroscope on biceps and wrist (non-ubiquitous)	Indirect/Error (sens, spec)
	Slowness (walking test speed)				Indirect/Assoc. (correlation)
	Weakness (grip strength)				Indirect/Assoc. (correlation)
50. Schwenk (2015) [86]	Frailty level (Fried’s: robust vs. prefrail vs. frail)	(a) gait patterns (b) balance (c) activity patterns	(a) no/no (b) no/no (c) yes/yes	(a) & (b) Multiple inertial wearable sensors LEGSys TM + BalanSens TM (non-ubiquitous) (c) PAMSys TM IMU on sternum (pocket) (on-body)	(a) (b) & (c) Indirect/error (AUC)
51. Regterschot (2015) [47]	Frailty level (Groningen Frailty Indicator)	STS transitions (transitions)	Yes/no	Philips pi-Node IMU (a) on chest (on-body) (b) on hip (on-body)	Indirect/Error (AUC)
	Performance (TUG)				Indirect/Assoc.
	Weakness (STS5 stopwatch)				Indirect/Assoc. (correlation)
52. Martínez-Ramírez (2015) [79]	Frailty level (Fried’s: robust vs. prefrail vs. frail)	Gait patterns	No/no	Mtx Xsense inertial sensor on lower back (on-body)	Indirect/Assoc. (ANOVA) Indirect/error (AUC)
53. Galán-Mercant & Cuesta-Vargas (2015) [102]	Frailty level (Fried’s: robust vs. prefrail/frail)	Up and go	No/no	iPhone 4 ^®^ IMU sensors on chest (on-body)	Indirect/Assoc. (*t*-test) Indirect/error (AUC)
54. Castro (2015) [94]	Frailty level (Fried’s: robust vs. prefrail/frail)	Activity patterns	Yes/yes	Smartphone accelerometer, GPS, NFC reader, microphone on waist (on-body)	Indirect/Assoc. (*t*-test)
55. Zhang (2014) [66]	Responsiveness (test-retest)	STS transitions (cycles)	No/no	3DoF accelerometer pendant on chest (on-body)	Direct/Error (test-retest: ICC, SEM)
56. Regterschot (2014) [53]	Responsiveness (before/after intervention)	STS transitions (transitions)	Yes/no	Philips pi-Node 9DoF IMU on hip. (ubiquitous)	Direct/Error (absolute SRM)
57. Millor (2014) [69]	Responsiveness (before/after intervention)	STS transitions (cycles)	No/no	MTx Xsense inetrial orientation tracker on lower back (on-body)	Direct/Assoc.
58. Galán-Mercant & Cuesta-Vargas (2014) [105]	Frailty level (Fried’s: robust vs. prefrail/frail)	Balance	No/no	iPhone 4 ^®^ accelerometer on chest (on-body)	Indirect/Assoc. (Mann-Whitney U-test)
59. Galán-Mercant & Cuesta-Vargas (2014) [103]	Frailty level (Fried’s: robust vs. prefrail/frail)	Up and go	No/no	iPhone 4 ^®^ accelerometer on chest (on-body)	Indirect/Assoc. (Mann-Whitney U-test)
60. Aguilar-Farías (2014) [95]	Physical activity (time active)	Activity patterns	Yes/yes	ActiGraph GT3X+ on waist (ubiquitous)	Direct/Error (sens, spec, accuracy, AUC)
61. Galán-Mercant & Cuesta-Vargas (2013) [48]	Frailty level (Fried’s: robust vs. prefrail/frail)	STS transitions (transitions)	Yes/no	iPhone 4 ^®^ accelerometer and gyroscope on chest (on-body)	Indirect/Assoc.
62. Chang (2013) [70]	Frailty level (Fried’s: robust vs. prefrail vs. frail)	Gait patterns	No/no	Pressure sensors in a chair (ubiquitous)	Indirect/Error (sens, spec)
		STS transitions (cycles)	No/no	Pressure sensor in a chair (ubiquitous)	
		Body weight	No/no	Pressure sensor in a chair (ubiquitous)	
		Balance	No/no	Pressure sensor on the floor (ubiquitous)	
		Questionnaire	No/no	Tablet (ubiquitous)	
63. Kaye (2012) [76]	Slowness (walking test speed)	Gait patterns	Yes/yes	Multiple MS16A X10 PIR sensors on ceiling (ubiquitous)	Direct/Assoc. (regression)
	Performance (Tinetti balance scale)				
	Weakness (CST)				
64. Ganea (2011) [49]	Frailty level (Fried’s: robust vs. prefrail/frail)	STS transitions (transitions)	Yes/no	IMU on chest (on-body)	Indirect/Error (sens, spec)
	Responsiveness (before/after intervention)				
65. Berke (2011) [96]	Physical activity (PA questionnaire)	Activity patterns	Yes/yes	Accelerometer, microphone, barometer, temperature, humidity, light on waist (ubiquitous)	Indirect/Assoc. (correlation)
66. Zijlstra (2010) [54]	Weakness (lower limbs peak power)	STS transitions (transitions)	Yes/no	Multiple 9DoF IMU on waist and chest (non-ubiquitous)	Direct/Error (RMS, correlation)
67. Nicolai (2010) [97]	Performance (SPPB)	Activity patterns	Yes/no	BioAGM Physilog accelerometer and gyroscope on chest (on-body)	Indirect/Assoc. (correlation)
